# Dual-sense slot-based CP MIMO antenna with polarization bandwidth reconfigurability

**DOI:** 10.1038/s41598-023-42569-1

**Published:** 2023-09-26

**Authors:** Niamat Hussain, Khaled Aljaloud, Rifaqat Hussain, Ali H. Alqahtani, Zia Ullah Khan, Farooq A. Tahir, Qammer H. Abbasi

**Affiliations:** 1 Department of Intelligent Mechatronics Engineering, Sejong University, Seoul, Korea; 2 College of Engineering, Muzahimiyah Branch, King Saud University, P.O. Box 2454, Riyadh, 11451 Saudi Arabia; 3 Antenna and Electromagnetics Research Group, School of Electronic Engineering and Computer Science, Queen Mary University of London, London, UK; 4 Department of Electronic and Electrical Engineering, The University of Sheffield, Sheffield, UK; 5 James Watt School of Engineering, University of Glasgow, Glasgow, UK

**Keywords:** Engineering, Physics

## Abstract

In this letter, a compact, planar circularly polarized (CP) sub-GHz slot-based multiple-input-multiple-output (MIMO) antenna with dual sense CP along with polarization bandwidth reconfigurability is presented. The pentagonal reactively loaded slot is fed by two folded tapered feedlines to achieve CP. The antenna offers left-hand-circular polarization (RHCP) with the as well as right hand circular polarization (LHCP). The antenna exhibit linearly polarization (LP) by exciting two ports simultaneously. Moreover, the antenna CP resonance can be reconfigured by varying the capacitance of the varactor diode. The antenna has a wide −10 dB operating frequency band from 578–929 MHz. while the axial ratio (AR) bandwidth ranges from 490–810 MHz. Moreover, the two elements MIMO are optimized and placed on compact dimensions 100 × 100 × 0.76 mm^3^ to realize pattern diversity. The antenna’s key characteristics are compact size, wide-band sub-GHz operation, dual sense CP, polarization bandwidth reconfigurability and good MIMO performance. Thus, it is a suitable candidate to be utilized in CubeSats applications in sub-GHz bands.

## Introduction

Circular polarized (CP) antenna offers many advantages over the linearly polarized (LP) antennas due to their decreased polarization mismatch losses, and ability to provide freedom of antenna orientation. Therefore CP antennas are desired for satellite applications, WiMax, WLAN, RFID-tags, and 5G applications. Several efforts have been put in the literature to design CP antennas^1^. Slit-slot and microstrip patch-based antennas offer bidirectional CP radiation, where the rotating senses of CP in the front and back sides are presented in^2–9^.

Moreover, various methods have been employed to achieve polarization reconfigurable antennas that offer right-handed circular polarization (RHCP) and left-handed circular polarization (LHCP). The ability to operate with both senses (RHCP and LHCP) enables frequency reuse and doubles the capacity of the communication system^10^. The CP senses can be reconfigured in single port antennas by switching the states of at least two p-i-n diodes^11–16^ and MEMS^17^. These kinds of antennas need additional complex biasing circuitry to control the diode, which increases the cost and reduces the antenna efficiency and CP bandwidth. Alternatively, CP sense reconfigurable antennas utilizing dual-ports have also been presented due to their wide-band characteristics^18–20^.

Most importantly, if the antenna can be switched between two senses of CP (RHCP and LHCP) and LP, as well as the operating frequency, it will allow the user to roam to virtually any existing communication network system. Therefore, some works have been reported to designing the frequency and polarization reconfigurable antennas^21–23^. In these designs, varactor and p-i-n diodes are utilized to achieve continuous frequency-polarization agility. At the same time, the switching between three polarization states and tunable working frequency bands are accomplished by microfluidic injection in^24^.

**Table 1:** Performance comparison with the state of the art antennas.

Ref.	Ant. type	Size reduction	Sub-GHz operation?	CP bandwidth reconfigurability	Polarization states bands	MIMO
^18^	Slot antenna	No	No	No	LHCP	No
^19^	Cut ring microstrip patch	No	No	No	LP, RHCP, LHCP	No
^20^	Slot, patch	No	No	NO	RHCP, LHCP	No
^21^	Patch antenna array	No	No	Yes	LP, RHCP, LHCP	No
^22^	Square patch	No	No	No	LPH- or V-polarization	No
^23^	Stub-loaded microstrip patch	No	No	Yes	LP, RHCP, LHCP	No
^24^	Slot antenna	Yes	No	Yes	LP, RHCP, LHCP	No
^25^	Slot antenna		Yes	NO	RHCP, LHCP	No
^26^	Horizontally meandered strip	Yes	Yes	No	CP	No
^27^	Patch antenna	Yes	Yes	No	CP	No
Prop. work	Yes	Yes	Yes	Yes	LP, RHCP, LHCP	Yes

It is worth noting that most of the CP antenna designs as discussed are operating at frequency bands above 1 GHz band. The design of CP antenna with the features of frequency-polarization reconfigurability with wide-band and compact size characteristics is always challenging at the sub-GHz spectrum. Although many CP antennas have been presented at the sub-GHz band for RFID tags, the internet of things, CubeSat, and several other applications^25–27^, they do not offer polarization bandwidth reconfigurability. Additionally, multiple-input-multiple-output (MIMO) configurations are essential for high data rates with seamless connectivity. None of the aforementioned CP antenna with reconfigurability has MIMO capabilities. The proposed polarization bandwidth reconfigurable antenna is compared with state-of-the-art CP antennas. The proposed design is compared with other reference CP designs as shown in Table 1. The proposed antenna design outperforms over its operating capability in the sub-GHz bands, polarization bandwidth reconfigurability, compact structure and MIMO configuration.

CP antennas have several advantages in sub-GHz wireless communication systems, particularly in CubeSat applications. These antennas improve signal propagation, reduce the impact of orientation, mitigate polarization mismatch, and find use in RFID, IoT, and satellite communication systems. The proposed antenna design is suitable for CubeSats operating in sub-GHz bands with CP characteristics. CP antennas are beneficial for satellite communication systems in the sub-GHz frequency range as they enhance signal reception and transmission, compensating for polarization mismatch caused by satellite orientation and ground station antennas. The specific frequency range of 578 to 929 MHz is mentioned as a potential range where circularly polarized antennas are commonly used. This range includes VHF and UHF bands, which are utilized by CubeSats, wireless microphone systems, UHF RFID systems, and certain satellite communication systems. Circularly polarized antennas help maintain consistent signal quality and improve performance in these applications.

This letter focuses on the design of a dual-port slot-based MIMO antenna with multiple polarization (LP, RHCP, LHCP) along with polarization bandwidth reconfigurable MIMO antenna. The novelty and unique features of the proposed antenna design are given below:

1. This letter focuses on the design of compact circularly polarized antenna for CubeSat applications. Most of the sub-GHz antenna for CubeSat operation are 3D structure which needs additional deployment mechanism. Thus, adding more complexity to the CubeSat design. However, the proposed antenna is low profile and planar and doesn’t need any additional deployment structure.
2. This work is based on dual-port slot-based antenna with multiple polarization (LP, RHCP, LHCP). A single antenna structure is being utilized to obtain 3 different types of CP at sub-GHz operation.
3. The proposed antenna design is optimized in MIMO configuration with good MIMO performance metrics.
4. The proposed antenna design has the 3-dB axial ratio (AR) bandwidth that can be tuned from 490–810 MHz by utilizing a single varactor diode. This is the prominent feature of the antenna design to provide switching flexibility between narrow-band and wide-band CP operation in wide-band antennas structure.
5. The 2-element MIMO antenna offers a good isolation within a compact size and stable radiation patterns.
6. To the best of the author’s knowledge, this design is the first of its kind that combines the advantages of compact size (100 mm × 100 mm × 0.76 mm), polarization bandwidth reconfigurability, narrow & wide bands CP configuration, and MIMO operation at sub-GHz bands.

The distinguishing features as described above show the uniqueness and appropriateness of the proposed antenna design to be utilized in CubeSat applications operating at sub-GHz bands.

## Antenna geometry

### Single element

**Figure 1:**
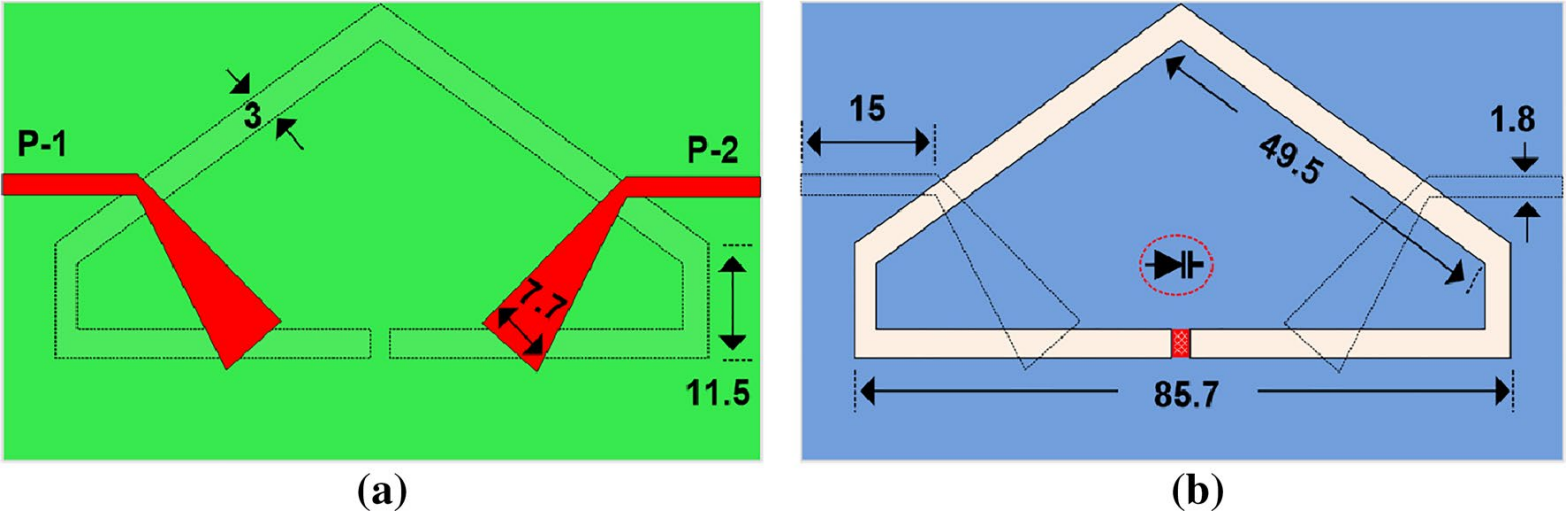
Single-element antenna (**a**) Bottom view (**b**) Top view, (units in mm).

The schematic diagram of the proposed dual-feed single-element antenna is shown in Fig. 1a and b. The antenna was etched on an FR-4 substrate board with thickness of 0.76 mm. The antenna consists of a pentagonal loop slot-line having a varactor diode in its center. The slot was fed at two corners (left and right side) with a folded tapered feedline. That is, Port-1 and Port-2 with the upper folded-slot make the single-element.

**Figure 2:**
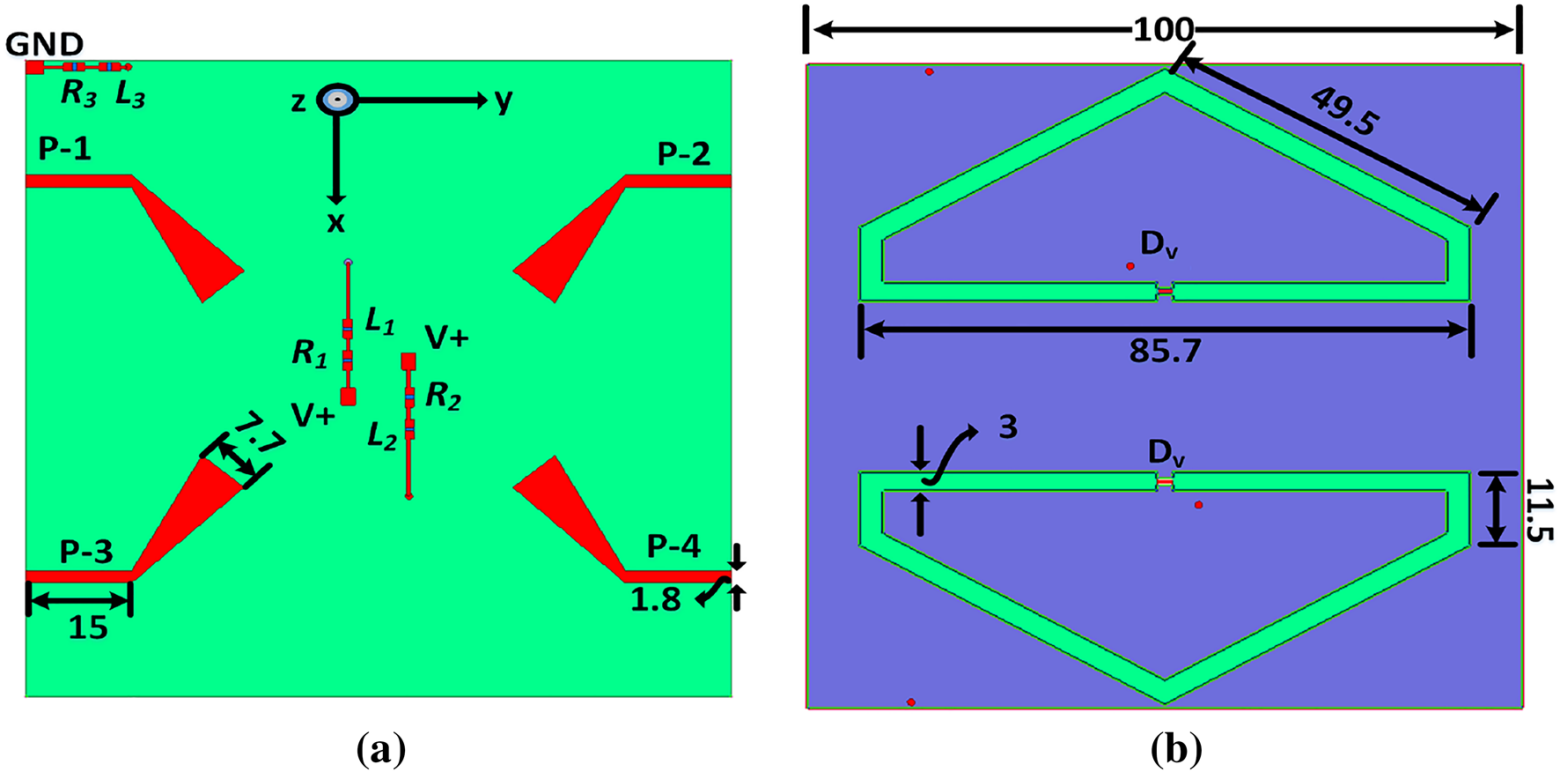
Proposed polarization bandwidth reconfigurable MIMO antenna: (**a**) bottom and (**b**) top view, (units in mm).

### Proposed MIMO antenna

**Figure 3:**
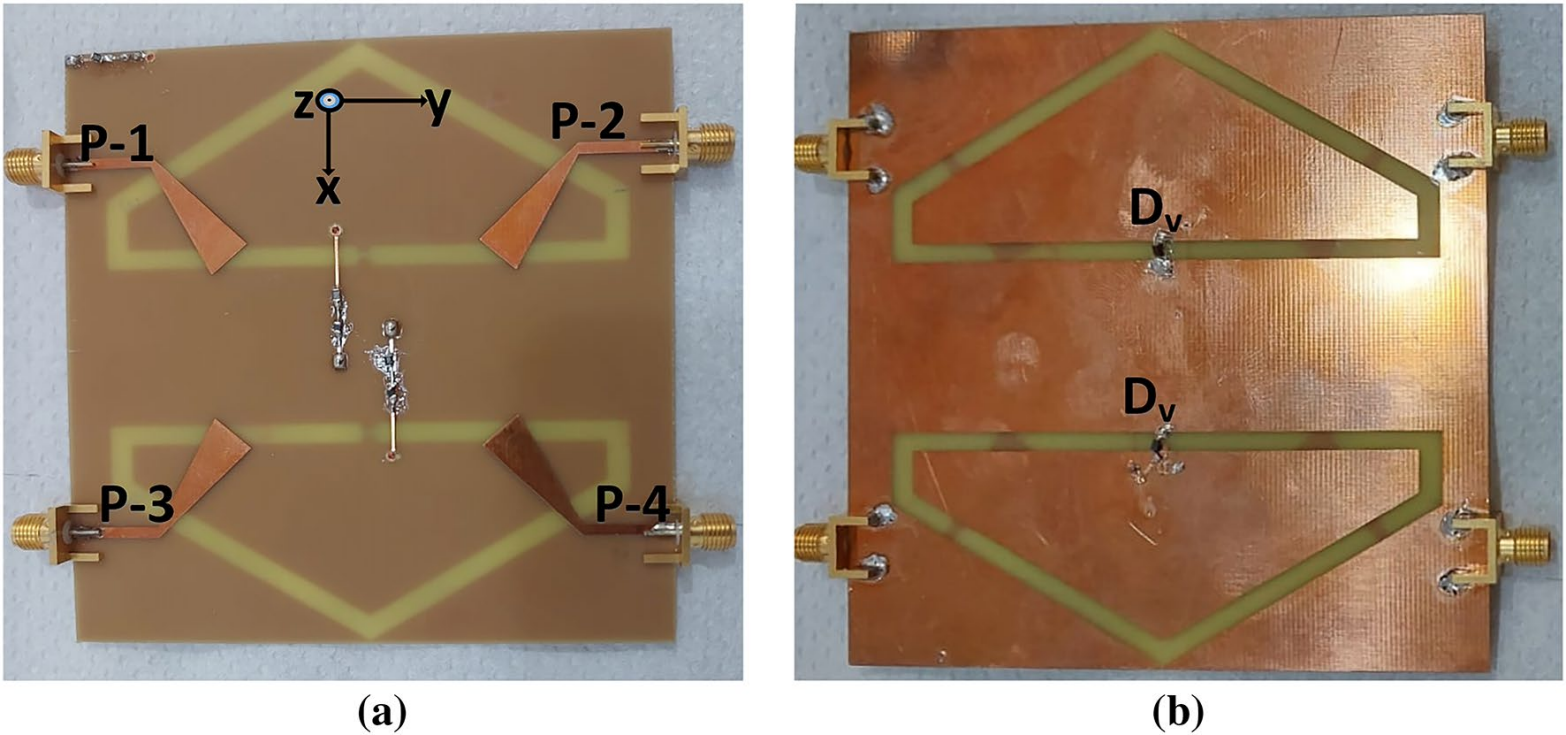
Fabricated antenna: (**a**) bottom view (**b**) top view.

The single element was then rotated along the axis with 360-degree for the MIMO configuration while keeping the parameters same of the antenna, as shown in Fig. 2. Both the antenna’s elements are closed spaced within the substrate size to achieve the desired MIMO performance metrics. To validate the design concept, a prototype of the antenna was fabricated and tested. The snaps of the prototype are shown in Fig. 3a and b. The varactor diode (SMV2019) was soldered and connected with the inbuilt circuitry (no additional circuit board is needed) for tuning the AR bandwidth.

### Operating mechanism

For the proposed antenna design, a sub-GHz band communications was selected due to its numerous advantages. The slot-based antenna design are quite popular due to its ease of manufacturing, integration with other circuit components, planar structure, wide-band attributes, and omni-directional radiation patterns, all of which are well suited for the proposed antenna design.The proposed slot has been configured in a meandering closed loop pattern to achieve compact size, resembling a non-uniform pentagonal shape as depicted in Fig. 1. The slot is energized using two transmission lines (TL): the left feed (Feed-1) for left-hand circular polarization (CP) and the right feed (Feed-2) for right-hand CP. The TLs have been optimized to achieve impedance matching bandwidth. The modified dimensions of the TLs are provided in Fig. 2a. Furthermore, the slot-line is augmented with a capacitor (Cap), illustrated in Fig. 2b, to further optimize the electrical dimensions of the slot and enhance impedance matching bandwidth.The final optimized configuration spans the frequency range of 578–929 MHz with capacitive loading.

Furthermore, a parametric analysis are performed to enhance performance in terms of size reduction and bandwidth expansion. The slot antenna is coupled with a capacitor (Cap), as shown in Fig. 2b. The capacitance values are varied to assess their impact on the antenna performance. It has been observed that 0.38 pF has achieved wide-band operation while other values resulted in different input impedance matching.

### CP generation mechanism

**Figure 4:**
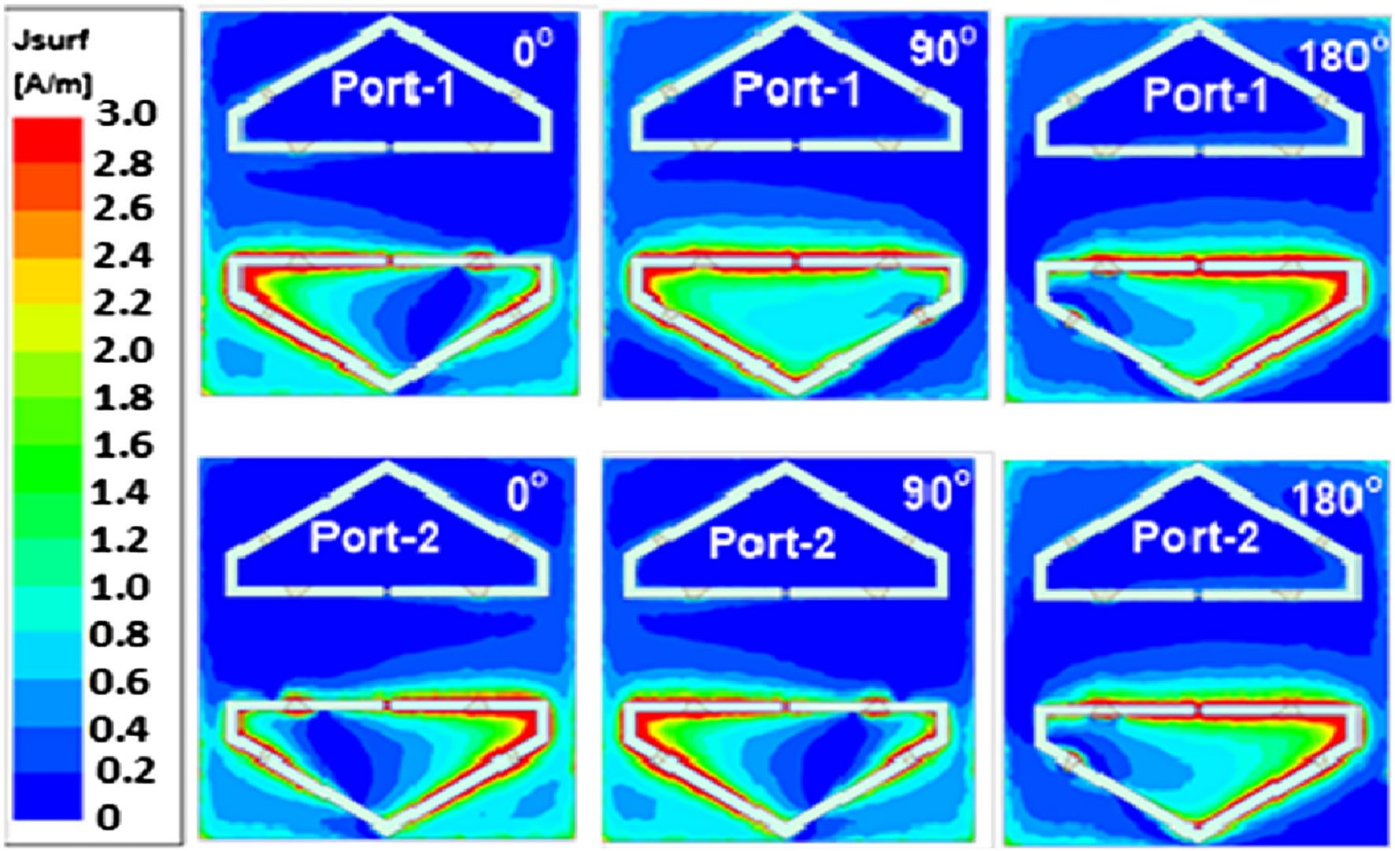
Rotating E-field of antenna: Port-1 and 2 (0.31 pF).

The E-field distribution on the antenna for two ports is investigated to explain the CP mechanism of the MIMO antenna (Fig. 4). For port-1, the E-field rotates in a clock-wise direction, giving the RHCP radiation. However, it turns in a counter clock-wise direction for port-2, which generates the LHCP. Moreover, it is also observed that there is a negligible effect on antenna 2 (no E-field is seen), during the excitation of ports 1 and 2, enabling the high isolation between the MIMO elements. The proposed antenna design envisioned to be integrated into the structure of the CubeSat during its manufacturing process. This integration could involve attaching the planar antenna to a suitable surface of the CubeSat, taking into account factors such as size, weight, and compatibility with other components.

To understand the circular polarization (CP) mechanism of the MIMO antenna with two ports, the distribution of the electric field (E-field) on the antenna is thoroughly investigated. This investigation aims to analyze the behavior of the E-field and its impact on the radiation characteristics of the antenna. Figure 4 provides a visual representation of the antenna structure and the observed E-field distribution. Specifically, when port-1 of the MIMO antenna is excited, it is observed that the E-field rotates in a clockwise direction. This clockwise rotation of the E-field results in the emission of right-hand circularly polarized (RHCP) radiation. RHCP refers to the polarization where the electric field vector rotates in a right-handed circular pattern as the electromagnetic wave propagates away from the antenna.

In contrast, when port-2 is excited, the E-field exhibits a counter-clockwise rotation. This counter-clockwise rotation of the E-field generates left-hand circularly polarized (LHCP) radiation. LHCP polarization involves the rotation of the electric field vector in a left-handed circular pattern as the wave propagates. One crucial observation made during this investigation is that during the excitation of ports 1 and 2, there is a negligible effect on antenna 2. In other words, no significant E-field is detected on antenna 2 when both ports 1 and 2 are excited simultaneously. This negligible effect leads to a high level of isolation between the MIMO elements. Isolation is an essential characteristic in MIMO (Multiple-Input Multiple-Output) systems, as it ensures that the signals transmitted from one antenna element do not interfere with the signals received by the other elements. The high isolation achieved in this MIMO antenna configuration helps minimize cross-interference and improves the overall performance and reliability of the system.

By investigating the E-field distribution and the resulting polarization characteristics of the MIMO antenna, a comprehensive understanding of the CP mechanism is obtained. This knowledge is valuable for designing and optimizing MIMO antenna systems in various applications, such as wireless communications, radar systems, and satellite communications, where efficient signal transmission and reception are essential.

## Simulation and measurement results

### S-parameters

**Figure 5:**
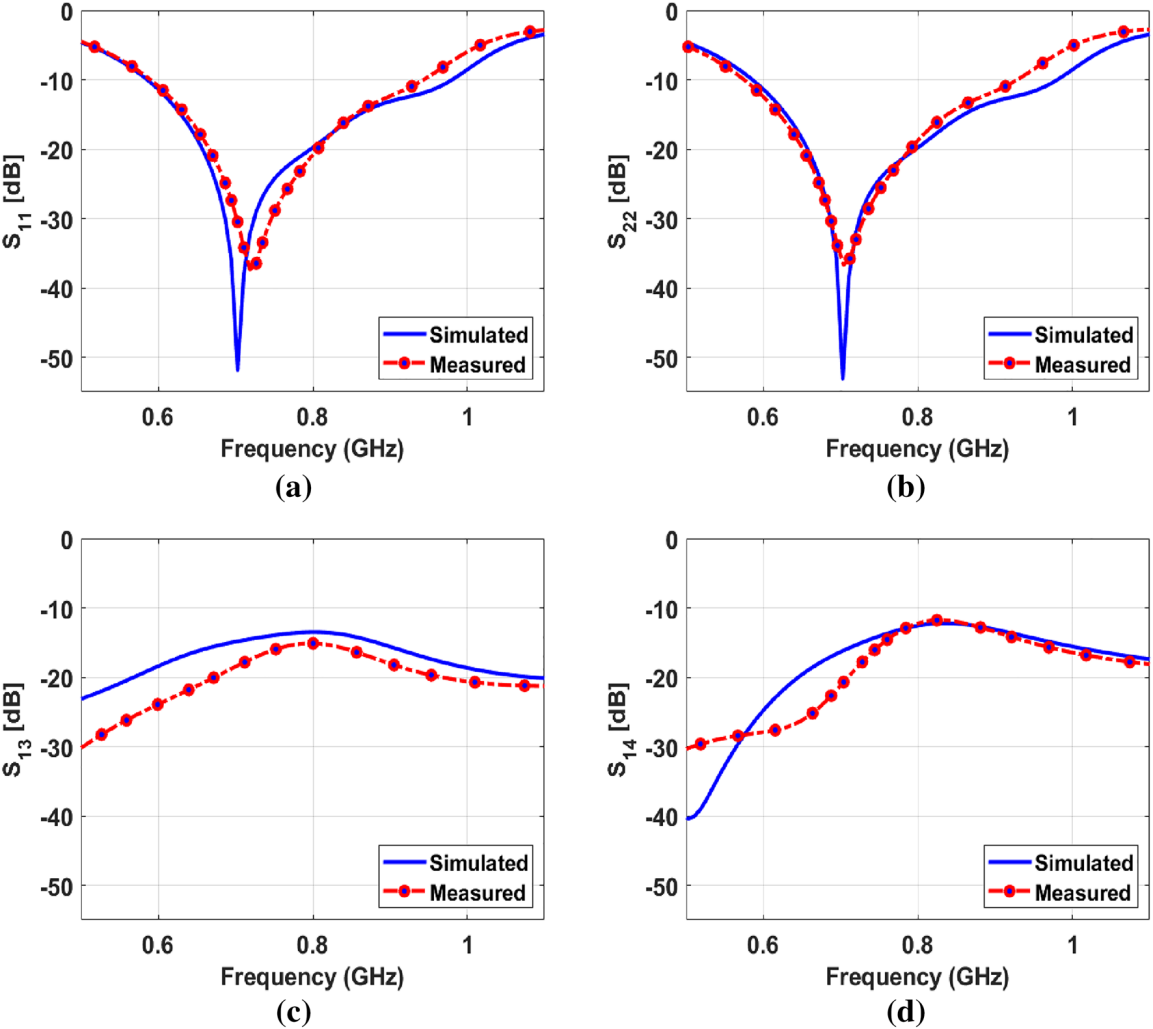
Reflection coefficient curves (**a**) $\vert$s$_{11}\vert$ (**b**) $\vert$s$_{22}\vert$ (**c**) $\vert$s$_{13}\vert$ (**d**) $\vert$s$_{14}\vert$.

The simulated and measured ($\vert$s_ii_ $\vert$ < − 10 dB) impedance matching curves for port-1 and port-2 is shown in Fig. 5a and b, respectively.

For brevity, the reflection coefficient curves for port-1 and port-2 are shown, as other ports show a similar response. Thanks to the unique capacitive loaded slot structure fed with a tapered feedline, the antenna offers a very wide measured impedance bandwidth ranging from 578–929 MHz for both port-1 and port-2. To the best of the author’s knowledge, this is the only design that offers such wide-band operation in a sub-GHz band within this compact size of the antenna with AR bandwidth reconfigurability.

The simulated and measured isolation among the MIMO elements in terms of the transmission coefficients for various ports are shown in Fig. 5c and d. Besides the minimal edge spacing of the antenna elements, it has a good isolation between the MIMO ports, which is found to be more than 11.75 dB for port-1 and port-4. While it is higher than 13.47 dB in the case of port-1 and port-3. The value is quite sufficient for the optimum MIMO performance.

### Axial ratio

**Figure 6:**
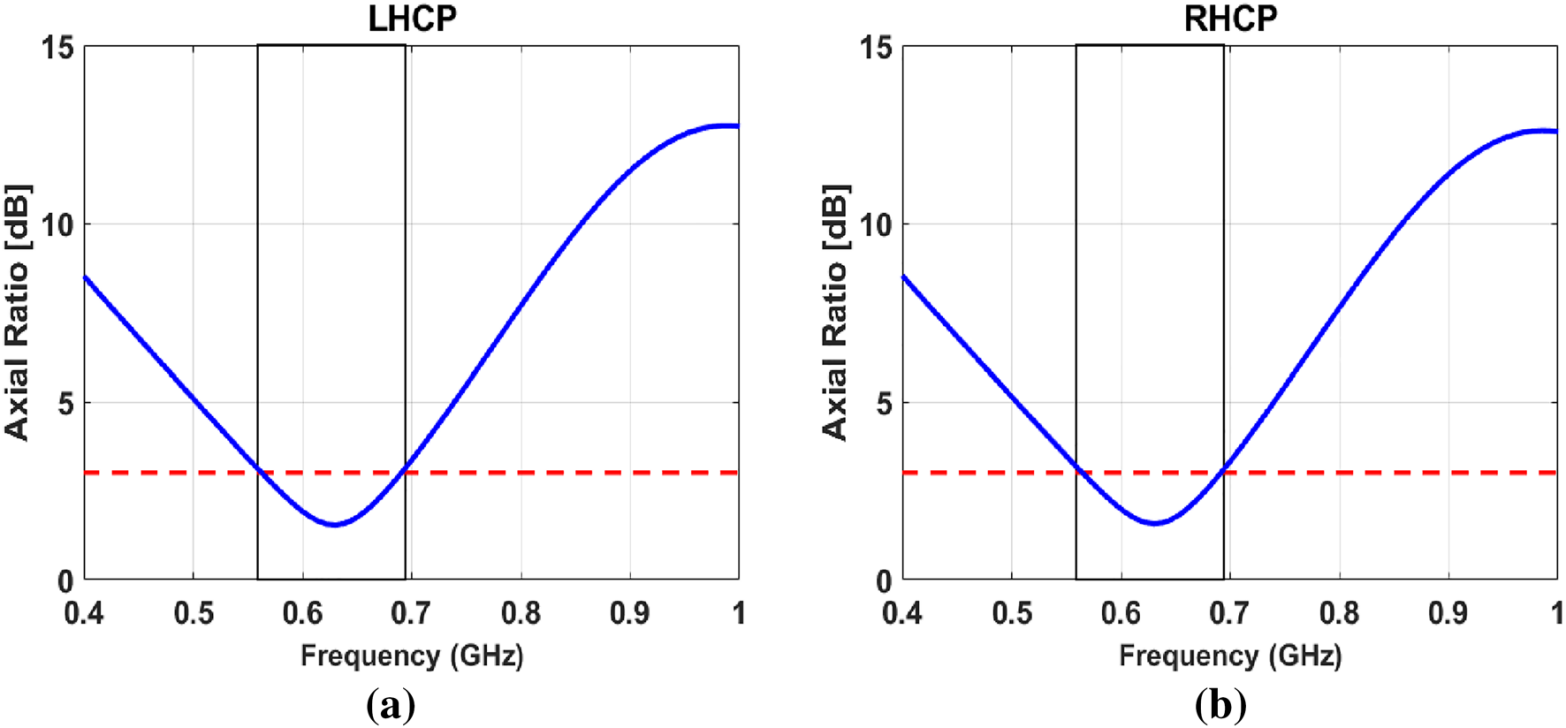
AR of the antenna − 0.31 pF (**a**) RHCP at port-1, (**b**) LHCP at port-2.

The unique feature of this design is that it offers dual sense CP (LHCP and RHCP). The antenna gives RHCP when port-1 (left port) is excited, while it gives LHCP when port-2 (right port) is excited. The simulated AR as a function of the frequency is shown in Fig. 6a and b. Both ports showed almost identical 3dB AR bandwidth. The antenna offers 3-dB LHCP bandwidth of 490–810 MHz, and RHCP bandwidth ranges from 493–811 MHz.

The proposed antenna design is capable of radiating in three different polarization modes: LP, RHCP, and LHCP. Unlike other antennas, this design doesn’t require any switching circuitry like p-i-n diodes or MEMS to switch between polarization modes. Typically, polarization reconfigurable antennas use switching mechanisms involving active components, which adds complexity and requires additional circuitry and control mechanisms.

In this antenna’s case, LP radiation is achieved by simultaneously feeding both ports (port-1 and port-2) with the desired signals. This results in LP radiation with an Axial Ratio (AR) value exceeding 40 dB. The Axial Ratio measures the circular polarization of the antenna’s radiation, with lower values indicating better circular polarization. By configuring the feeding of the antenna ports appropriately, LP radiation is achieved without the need for switching circuits. This sets it apart from other antennas that rely on additional components for polarization switching.

Overall, this antenna design offers the advantage of radiating in different polarization modes (LP, RHCP, and LHCP) without the complexity and potential drawbacks of switching circuitry. It provides versatility in terms of polarization selection and bandwidth reconfiguration, making it suitable for various applications.

**Figure 7:**
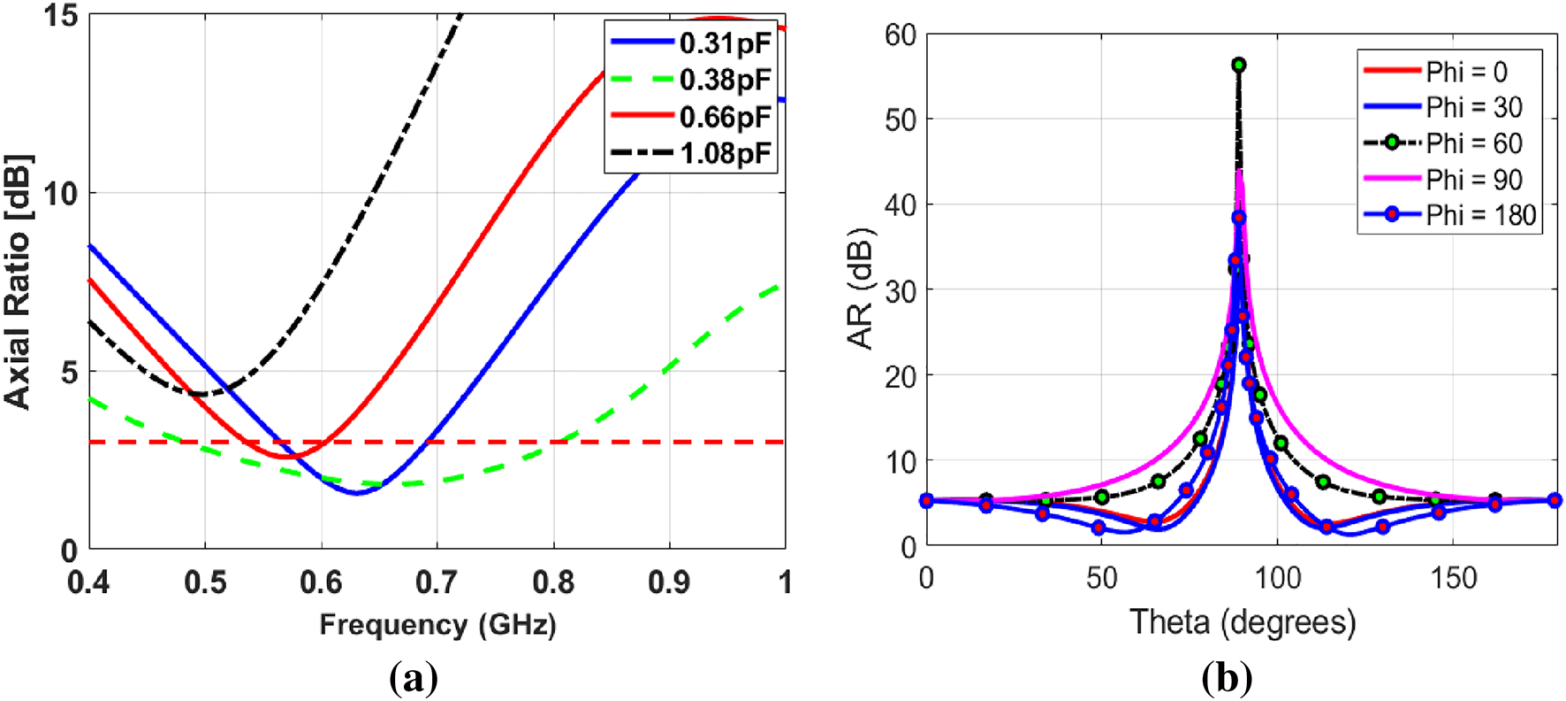
AR curves (**a**) Bandwidth reconfigurability (**b**) AR vs theta.

The antenna also has the additional advantage of AR bandwidth reconfigurability. The central frequency of the AR can be tuned by varying the capacitance of the diode. The resonance of AR can be shifted from 490 to 810 MHz by changing the capacitance values of varactor diode As shown in Fig. 7a. Various curves for capacitance values of 0.31 pF, 0.38 pF, 0.66 pF, and 1.08 pF are shown in given figure. It is clear that the AR can be tuned as wide-band as well arrow-band operation and can be shifted using the various values of capacitances. AR vs theta is plotted as shown in Fig. 7b.

### Far field characteristics

**Figure 8:**
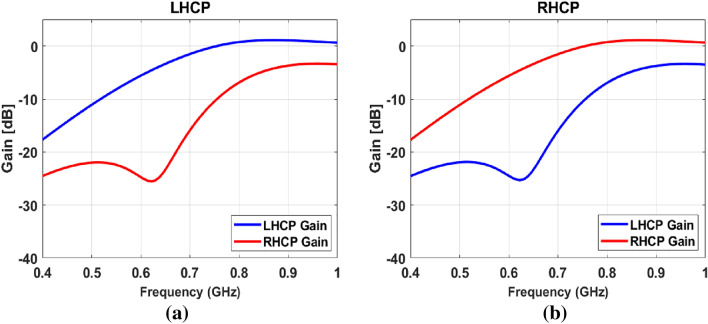
Gain of the antenna (0.31 pF) (**a**) LHCP at port-1, (**b**) RHCP at port-2.

The gain of the antenna for port-1 and port-2 (LHCP and RHCP gain) plots are shown in Fig. 8a and b. For port-1, the antenna RHCP gain dominates over LHCP, and its values reach up to 1.079 dBi within the operating band. Contrary to this, the LHCP dominates over RHCP for port-2, with a maximum value of 1.078 dBi. Moreover, the polarization isolation (difference between LHCP and RHCP for a particular port) is more than 12 dB.

**Figure 9:**
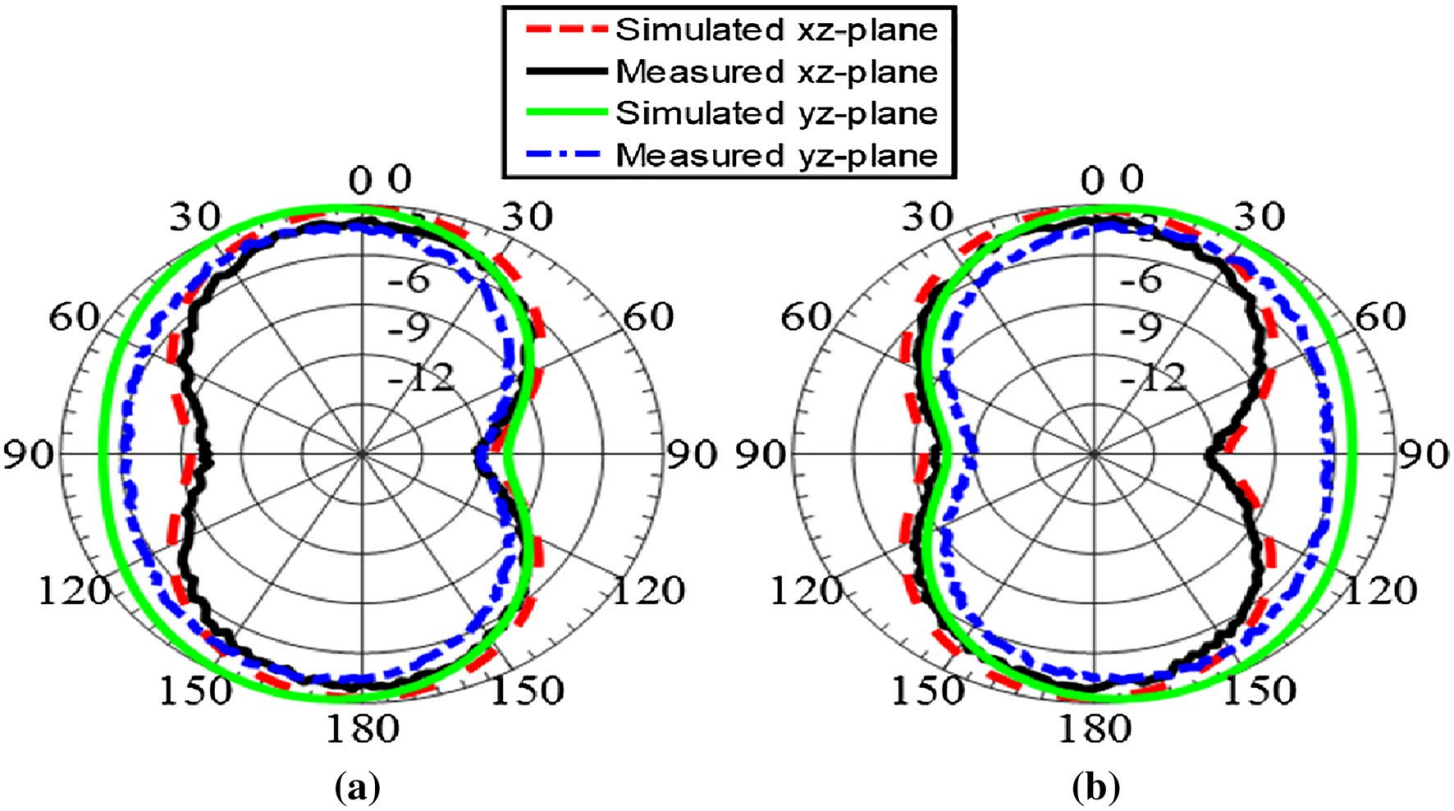
Normalized radiation patterns of the proposed antenna at 650 MHz (0.31 pF) (**a**) Port 1 (**b**) Port 2.

The radiation patterns for each polarization scenario are also shown in Fig. 9a and b. The antenna offers stable and bidirectional radiation patterns for the polarization states. The antenna provides a radiation pattern of conventional slot antenna, omni-directional at one plane and dumbbell shape at another plane. The simulated and measured antenna efficiency values are more than 78% over the entire band of operation.

**Figure 10:**
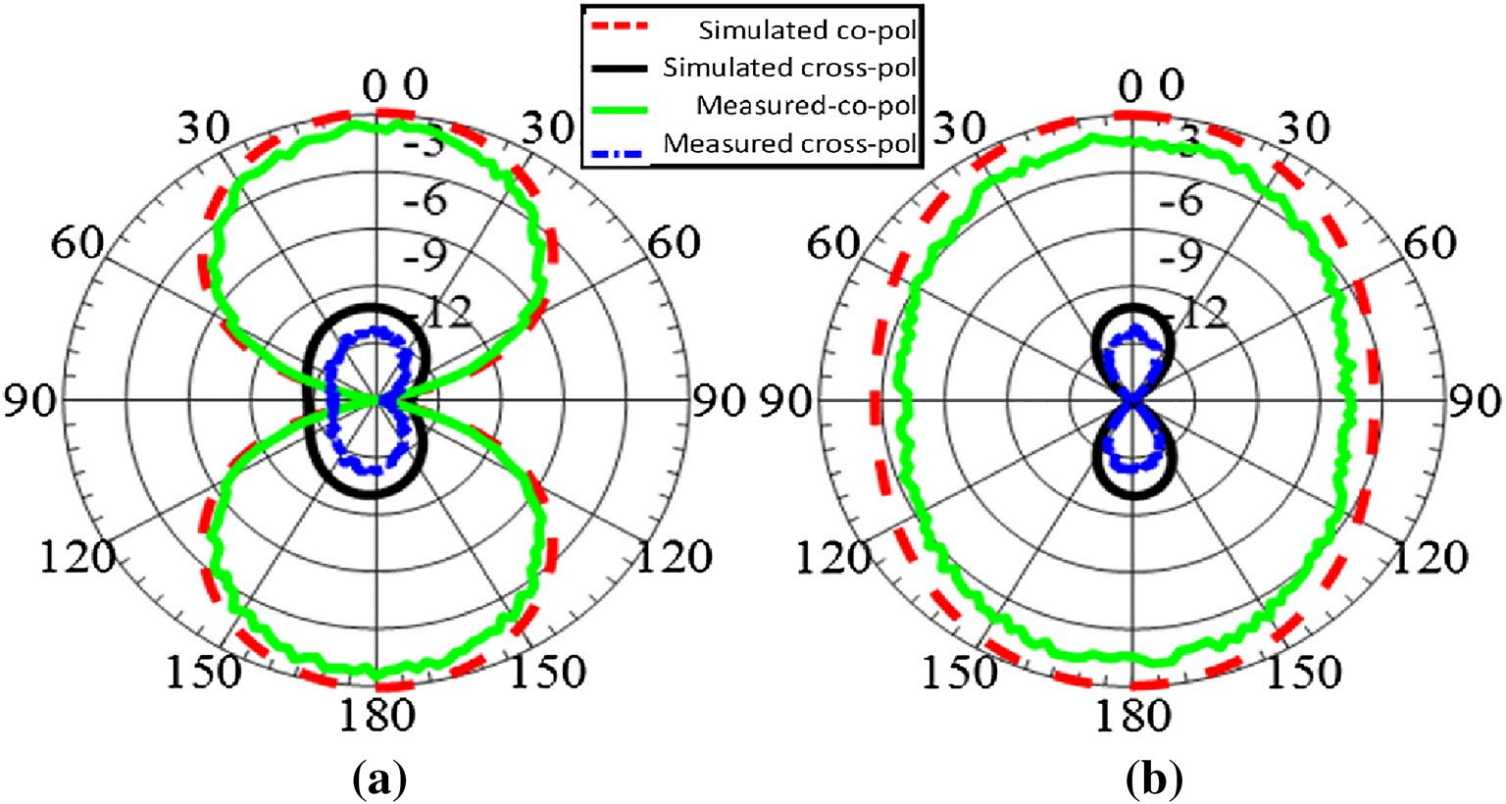
Normalized Co-pol and Cross-pol radiation at 650 MHz (0.31 pF) (**a**) xz-plane (**b**) yz-plane.

The proposed MIMO antenna is also investigated for co-pol and cross-pol patterns at 650 MHz as shown in Fig. 10. The radiation patterns show good polarization purity for slot-based antenna design with omni-directional radiation characteristics. The patterns shows good cross-pol discrimination of at least 14 dB. Hence, it can be concluded that the proposed antenna design exhibit good CP characteristics over the given band of interest.

The proposed antenna design exhibited omni-directional radiation patterns and providing 360-degree coverage pattern in the azimuth plane, thus enabling communication with multiple ground stations without requiring precise pointing or alignment. This can be particularly beneficial for CubeSats operating in low earth orbit, due to limited resources or maneuvering capabilities.

### MIMO diversity (ECC)

The diversity of the MIMO antenna in terms of envelope correlation coefficient (ECC) is calculated to show how much antenna elements are independent in their performance. The values are found to be very low, less than 0.02, ideal for the MIMO operation.

### Performance comparison

The proposed polarization-frequency reconfigurable antenna is compared with state-of-the-art antennas in terms of their typical functionalities. Most of the antennas^18–24^ do not offer sub-GHz operation and only a few designs^21–24^ offer frequency reconfigurability. Although most of the antennas are offering polarization reconfigurability, none of them have the extra advantages of the MIMO configuration. Thus, the proposed antenna outperforms its competitor designs with its operating capability in the sub-GHz band, polarization bandwidth reconfigurability, and MIMO configuration in a single design.

## Conclusions

A low-profile 2-elements MIMO antenna at sub-GHz with polarization-frequency agility is presented. The primary single-element antenna consists of a pentagonal slotline having a varactor diode in its center to obtain a compact antenna design. The unique capacitive loaded slot-line with the folded feedlines gives a broadband impedance bandwidth of 46.58% (578   929 MHz), and a 3dB AR bandwidth from 490 to 810 MHz. The proposed dual-feed antenna offers RHCP as well as LHCP radiations. Additionally, the AR bandwidth can be reconfigured by varying the capacitance of the varactor diode. The AR can be tuned over the operating bandwidth of 490 to 810 MHz. Furthermore, the antenna gives a high RHCP and LHCP gain of 1.079 dBi, and 1.078 dBi, respectively. The two-element MIMO antenna is realized on compact board size of 100 mm × 100 mm. The attractive features of this design are compact size and wide operating bandwidth in the sub-GHz band, polarization-bandwidth reconfigurability, and good isolation.

## Data Availability

All the necessary data to assess the outcomes of this study is presented within the this paper. Any supplementary data pertaining to this research can be inquired from the corresponding author.
